# Supplementary material for: A Decrease in Fatty Acid Synthesis Rescues Cells with Limited Peptidoglycan Synthesis Capacity
Source: mBio. 2023 Apr 5;14(2):e00475-23. doi: 10.1128/mbio.00475-23 (PMC10128001; doi:10.1128/mbio.00475-23)
Supplement: TABLE S1 [file mbio.00475-23-s0008.docx]

**SUPPLEMENTAL TABLES**

**Table S1. Pathways and Genes Relevant to this Study**

| **Gene(s)** | **Function** | **Significance in work** | **Relevant References** |
| --- | --- | --- | --- |
|  | **Fatty Acid Synthesis (FAS)** |  |  |
| *fapR* | Repressor of fatty acid synthesis; de-repression elicited by binding of malonyl-CoA | Deletion of *fapR* deregulates expression of *fapR* regulon; used as a control for *fapR** phenotypes | (1) (2) |
| *fapR** | Super-repressor allele of FapR | Represses FAS to rescue PG-limited cells | This study |
| *accABCD* | acetyl-CoA carboxylase (ACC) complex; carboxylates acetyl-CoA to malonyl-CoA | suppressor mutations rescue viability of PG-limited cells | (3) and This study |
| *fabF* | FAS II condensing enzyme; elongates fatty acids by 2 carbons; negatively regulated by FapR | Target of cerulenin (CER) | (4) |
| *fabHA* | FAS II condensing enzyme; required for initiation of fatty acid synthesis in non-stressed cells; negatively regulated by FapR | Proxy for transcription of *fapR* regulon | (5) |
|  | **Class A PBPs (aPBPs); PG synthesis** |  |  |
| *ponA* | major bifunctional glycosyltransferase/transpeptidase | Deletion is synthetic lethal in combination with either *ecsA, rasP,* or ponA | (6-8) |
| *pbpD, pbpF, and pbpG* | minor bifunctional glycosyltransferase/transpeptidase | These genes are deleted (together with *ponA*) in the Δ4 aPBP strain | (9) |
|  | **Elongasome; PG synthesis** |  |  |
| *rodA* | SEDS family monofunctional glycosyltransferase; part of the elongasome; essential for cell wall elongation | RodA provides transglycosylase (TG) activity to the elongasome | (10, 11) |
| *pbpH, pbpH* | Class B PBPs provide transpeptidase (TP) actvity | Provide transpeptidase (TP) activity to the elongasome | (12) |
| *rodZ* | Part of the elongasome; substrate of PrkC lipid II-sensing protein kinase | May serve to regulate the density of MreB filaments and thereby growth rate | (13) |
| *mreB* | cytoskeletal actin-like protein; helps organize elongasome function during cell wall elongation | MreB functions together with other MreB paralogs (MreBH, Mbl) | (14-16) |
| *mbl* | An MreB like protein (paralog). |  | (17) |
| *mreBH* | cytoskeletal actin-like protein; member of elongasome together with other MreB paralogs | Loss of *sigI* activation reduces *lytE/mreBH* expression | (6, 18) |
| *lytE* | Major cell wall autolysin; D,L-endopeptidase; regulated by SigI | Loss of *sigI* activation reduces *lytE/mreBH* expression | (6, 18, 19) |
| *mreC, mreD* | Membrane proteins functioning as part of the elongasome complex | The *mreBCD* operon is up-regulated by σ^M^ | (20, 21) |
|  | **WalKR: essential two-component system (TCS)** |  |  |
| *walK* | TCS sensor kinase; activates WalR | Mutations in *walK* rescue PG-limited cells via upregulation of *lytE* and *mreBH* | (6, 19) |
| *walR* | TCS response regulator; regulates *sigI* and autolysins including and the *mreBH/lytE* complex | WalH suppressor mutations restore viability of *sigI ponA* strains | (18) |
| *walH* | Negative regulator or WalKR system | Mutations in *walH* rescue PG-limited cells | (22) and this this study |
|  | **σ^M^ cell envelope stress response (CESR)** |  |  |
| *sigM* | ECF-type sigma factor; active in response to disruption of peptidoglycan synthesis to upregulate elongasome components and PG synthesis enzymes | σ^M^ regulon includes: *ponA, mreBCD, rodA,* and lipid II flippases (*amj, yngC*), and many other genes | (20, 23) |
|  | **σ^I^ stress response (EcsA, RasP dependent)** |  |  |
| *ecsA* | ATP-binding component of an ABC transporter; essential for the function of RasP | Deletion limits elongasome activity via loss of *sigI* activation; synthetic lethal with *ponA* | (6, 24) |
| *rasP* | Site II intramembrane protease; function necessary for the cleavage of FtsL, RsgI, RsiV, RsiW | Deletion limits elongasome activity via loss of *sigI* activation; synthetic lethal with *ponA* | (6, 25, 26) |
| *rsgI* | Anti-sigma factor; cleaved by RasP and regulated by an intrinsically disordered domain |  | (7) |
| *sigI* | sigma factor; control of heat shock response; regulation of elongasome | Deletion limits elongasome activity via loss of *sigI* activation; synthetic lethal with *ponA* | (6, 7) |
|  | **σ^W^ stress response (EcsA, RasP dependent)** |  |  |
| *rsiW* | Anti-sigma factor for σ^W^; Cleaved by PrsW (site 1) and RasP (site 2) protease | Controls a large regulon induced primarily by membrane-associated stresses | (23, 27-29) |
| *sigW* | ECF-type sigma factor; required for adaptation to membrane active agents | Modulates membrane fluidity in response to membrane active agents via regulation of *floA/T* and *fabHAF* | (23, 30) |

**SUPPLEMENTAL REFERENCES**

1. Schujman GE, de Mendoza D. 2008. Regulation of type II fatty acid synthase in Gram-positive bacteria. Curr Opin Microbiol 11:148-52.

2. Schujman GE, Guerin M, Buschiazzo A, Schaeffer F, Llarrull LI, Reh G, Vila AJ, Alzari PM, de Mendoza D. 2006. Structural basis of lipid biosynthesis regulation in Gram-positive bacteria. Embo j 25:4074-83.

3. Cronan JE. 2021. The Classical, Yet Controversial, First Enzyme of Lipid Synthesis: Escherichia coli Acetyl-CoA Carboxylase. Microbiol Mol Biol Rev 85:e0003221.

4. Schujman GE, Altabe S, de Mendoza D. 2008. A malonyl-CoA-dependent switch in the bacterial response to a dysfunction of lipid metabolism. Mol Microbiol 68:987-96.

5. Choi KH, Heath RJ, Rock CO. 2000. beta-ketoacyl-acyl carrier protein synthase III (FabH) is a determining factor in branched-chain fatty acid biosynthesis. J Bacteriol 182:365-70.

6. Patel Y, Zhao H, Helmann JD. 2020. A regulatory pathway that selectively up-regulates elongasome function in the absence of class A PBPs. Elife 9.

7. Brunet YR, Habib C, Brogan AP, Artzi L, Rudner DZ. 2022. Intrinsically disordered protein regions are required for cell wall homeostasis in *Bacillus subtilis*. Genes Dev 36:970-984.

8. Straume D, Piechowiak KW, Kjos M, Håvarstein LS. 2021. Class A PBPs: It is time to rethink traditional paradigms. Mol Microbiol 116:41-52.

9. McPherson DC, Popham DL. 2003. Peptidoglycan synthesis in the absence of class A penicillin-binding proteins in *Bacillus subtilis*. J Bacteriol 185:1423-31.

10. Meeske AJ, Riley EP, Robins WP, Uehara T, Mekalanos JJ, Kahne D, Walker S, Kruse AC, Bernhardt TG, Rudner DZ. 2016. SEDS proteins are a widespread family of bacterial cell wall polymerases. Nature 537:634-638.

11. Emami K, Guyet A, Kawai Y, Devi J, Wu LJ, Allenby N, Daniel RA, Errington J. 2017. RodA as the missing glycosyltransferase in *Bacillus subtilis* and antibiotic discovery for the peptidoglycan polymerase pathway. Nat Microbiol 2:16253.

12. Wei Y, Havasy T, McPherson DC, Popham DL. 2003. Rod shape determination by the *Bacillus subtilis* class B penicillin-binding proteins encoded by *pbpA* and *pbpH*. J Bacteriol 185:4717-26.

13. Sun Y, Hürlimann S, Garner E. 2023. Growth rate is modulated by monitoring cell wall precursors in *Bacillus subtilis*. Nature Microbiology doi:10.1038/s41564-023-01329-7.

14. Dersch S, Reimold C, Stoll J, Breddermann H, Heimerl T, Defeu Soufo HJ, Graumann PL. 2020. Polymerization of *Bacillus subtilis* MreB on a lipid membrane reveals lateral co-polymerization of MreB paralogs and strong effects of cations on filament formation. BMC Mol Cell Biol 21:76.

15. Schirner K, Errington J. 2009. Influence of heterologous MreB proteins on cell morphology of *Bacillus subtilis*. Microbiology (Reading) 155:3611-3621.

16. Kawai Y, Asai K, Errington J. 2009. Partial functional redundancy of MreB isoforms, MreB, Mbl and MreBH, in cell morphogenesis of *Bacillus subtilis*. Mol Microbiol 73:719-31.

17. Schirner K, Errington J. 2009. The cell wall regulator sigmaI specifically suppresses the lethal phenotype of *mbl* mutants in *Bacillus subtilis*. J Bacteriol 191:1404-13.

18. Huang WZ, Wang JJ, Chen HJ, Chen JT, Shaw GC. 2013. The heat-inducible essential response regulator WalR positively regulates transcription of *sigI, mreBH* and *lytE* in *Bacillus subtilis* under heat stress. Res Microbiol 164:998-1008.

19. Dobihal GS, Brunet YR, Flores-Kim J, Rudner DZ. 2019. Homeostatic control of cell wall hydrolysis by the WalRK two-component signaling pathway in *Bacillus subtilis*. Elife 8.

20. Eiamphungporn W, Helmann JD. 2008. The *Bacillus subtilis* sigma(M) regulon and its contribution to cell envelope stress responses. Mol Microbiol 67:830-48.

21. Zhao H, Patel V, Helmann JD, Dörr T. 2017. Don't let sleeping dogmas lie: new views of peptidoglycan synthesis and its regulation. Mol Microbiol 106:847-860.

22. Szurmant H, Bu L, Brooks CL, 3rd, Hoch JA. 2008. An essential sensor histidine kinase controlled by transmembrane helix interactions with its auxiliary proteins. Proc Natl Acad Sci U S A 105:5891-6.

23. Helmann JD. 2016. Bacillus subtilis extracytoplasmic function (ECF) sigma factors and defense of the cell envelope. Curr Opin Microbiol 30:122-132.

24. Heinrich J, Lundén T, Kontinen VP, Wiegert T. 2008. The *Bacillus subtilis* ABC transporter EcsAB influences intramembrane proteolysis through RasP. Microbiology (Reading) 154:1989-1997.

25. Parrell D, Zhang Y, Olenic S, Kroos L. 2017. *Bacillus subtilis* Intramembrane Protease RasP Activity in Escherichia coli and In Vitro. J Bacteriol 199.

26. Liu TY, Chu SH, Hu YN, Wang JJ, Shaw GC. 2017. Genetic evidence that multiple proteases are involved in modulation of heat-induced activation of the sigma factor SigI in *Bacillus subtilis*. FEMS Microbiol Lett 364.

27. Asai K. 2018. Anti-sigma factor-mediated cell surface stress responses in *Bacillus subtilis*. Genes Genet Syst 92:223-234.

28. Ho TD, Ellermeier CD. 2012. Extra cytoplasmic function sigma factor activation. Curr Opin Microbiol 15:182-8.

29. Helmann JD. 2006. Deciphering a complex genetic regulatory network: the *Bacillus subtilis* sigmaW protein and intrinsic resistance to antimicrobial compounds. Sci Prog 89:243-66.

30. Kingston AW, Subramanian C, Rock CO, Helmann JD. 2011. A σW-dependent stress response in *Bacillus* *subtilis* that reduces membrane fluidity. Mol Microbiol 81:69-79.
