# Supplementary figures and images for: A Decrease in Fatty Acid Synthesis Rescues Cells with Limited Peptidoglycan Synthesis Capacity
Source: mBio. 2023 Apr 5;14(2):e00475-23. doi: 10.1128/mbio.00475-23 (PMC10128001; doi:10.1128/mbio.00475-23)

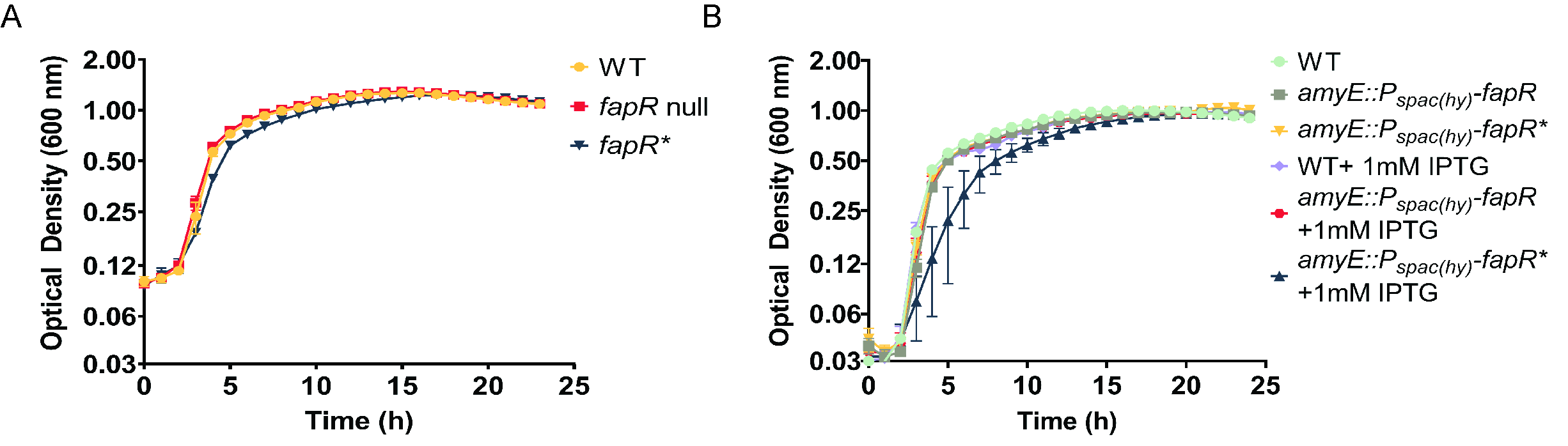

Supplement: FIG S1 [file mbio.00475-23-s0001.tif]

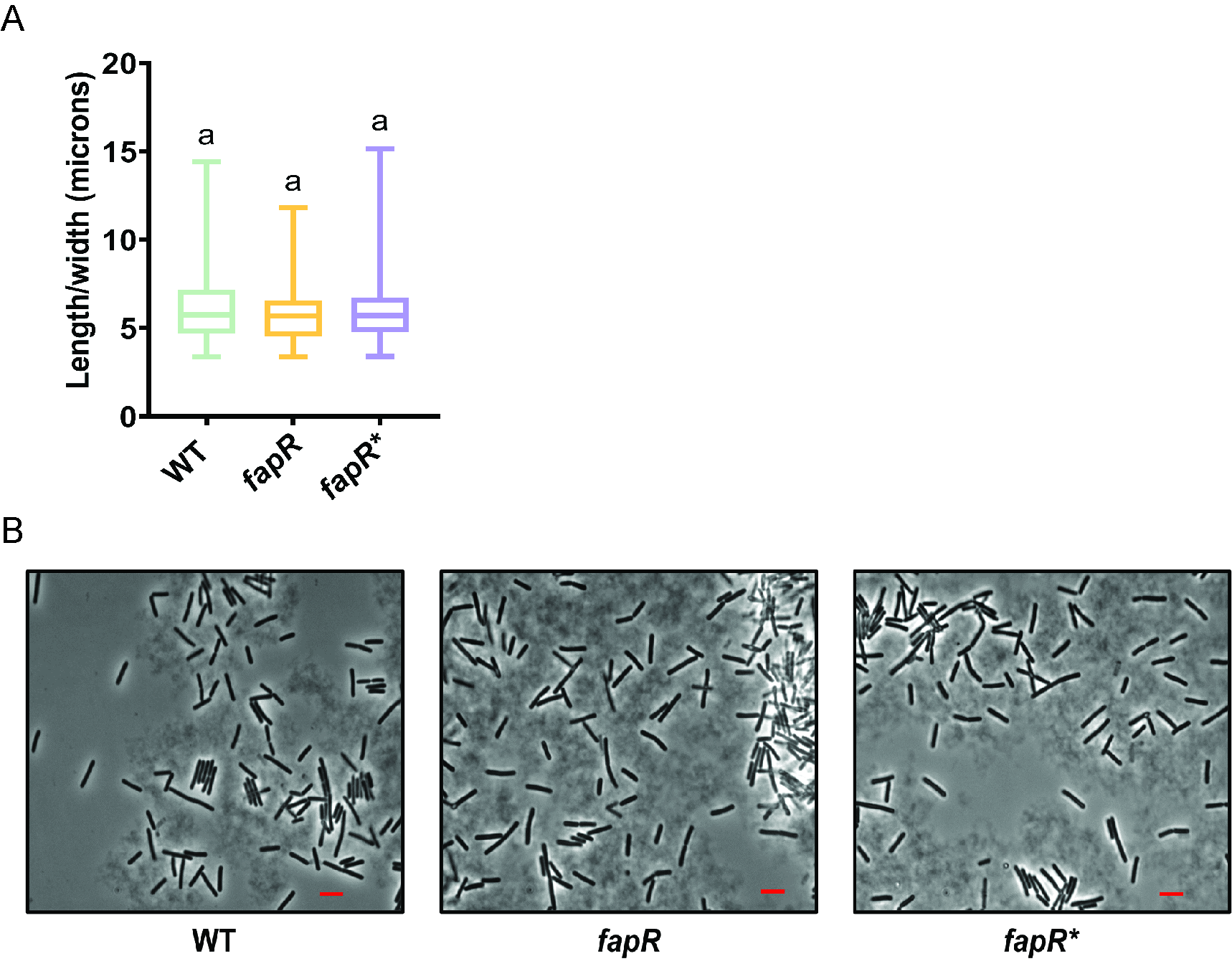

Supplement: FIG S2 [file mbio.00475-23-s0002.tif]

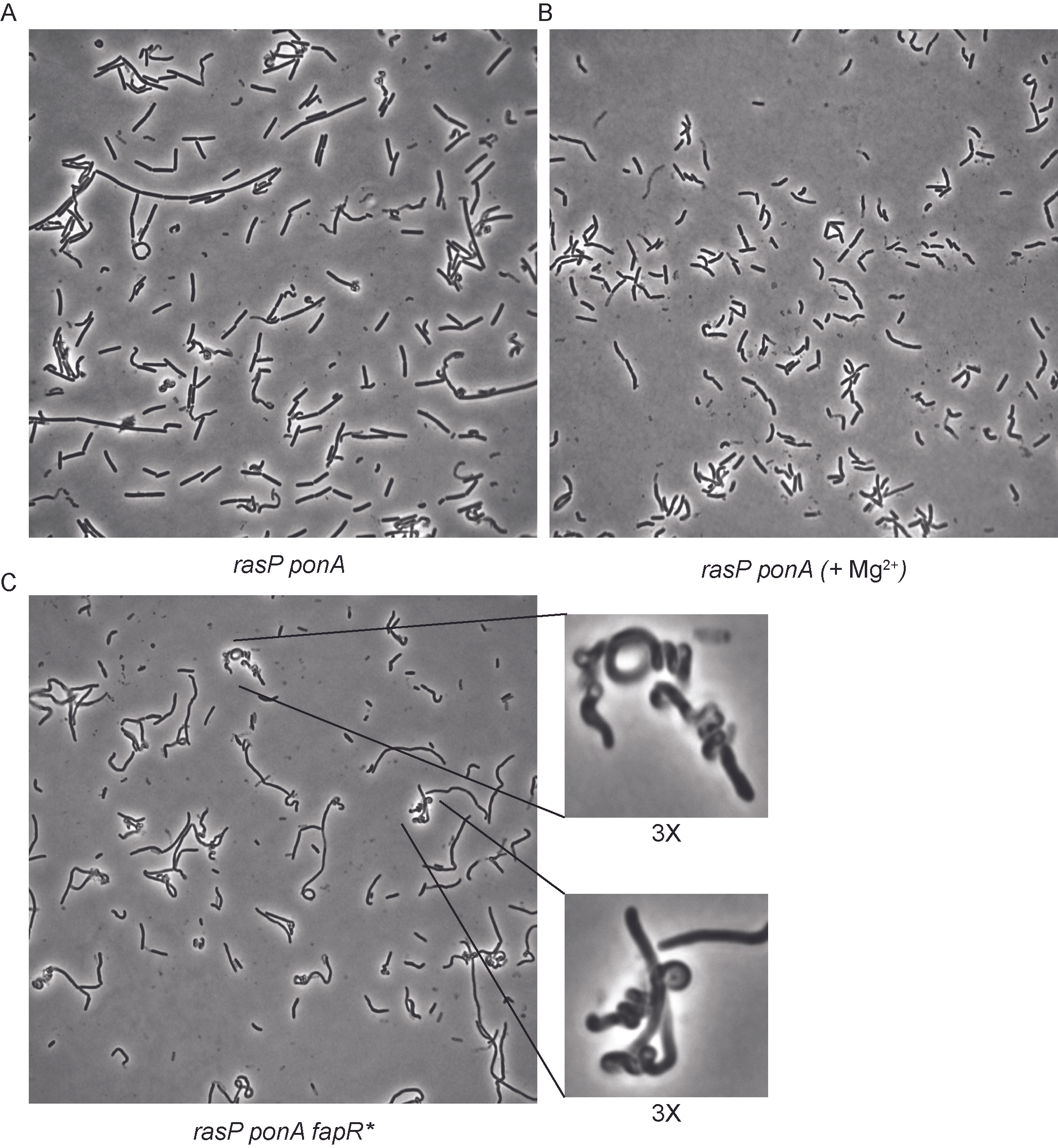

Supplement: FIG S3 [file mbio.00475-23-s0003.tif]

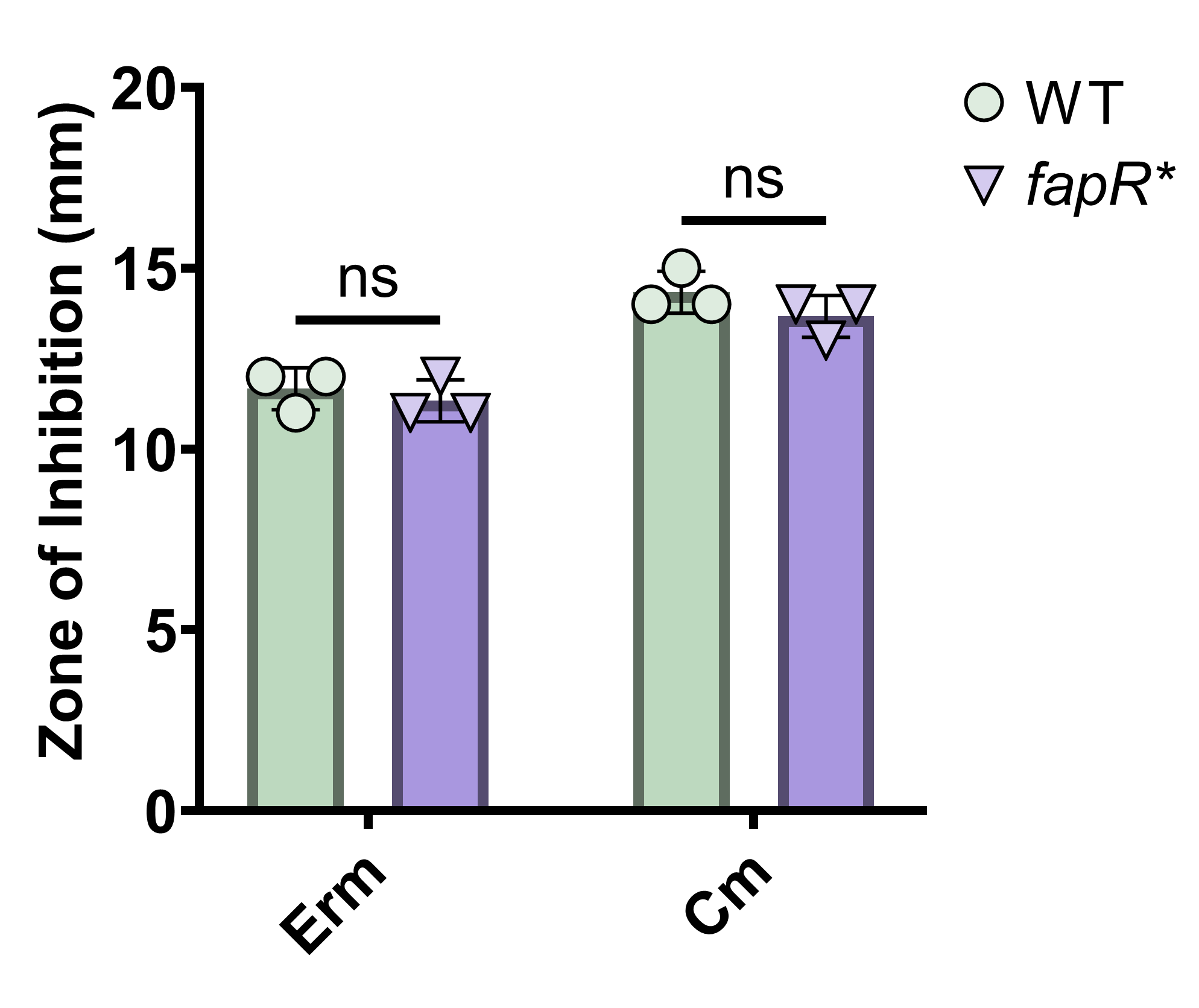

Supplement: FIG S4 [file mbio.00475-23-s0004.tif]

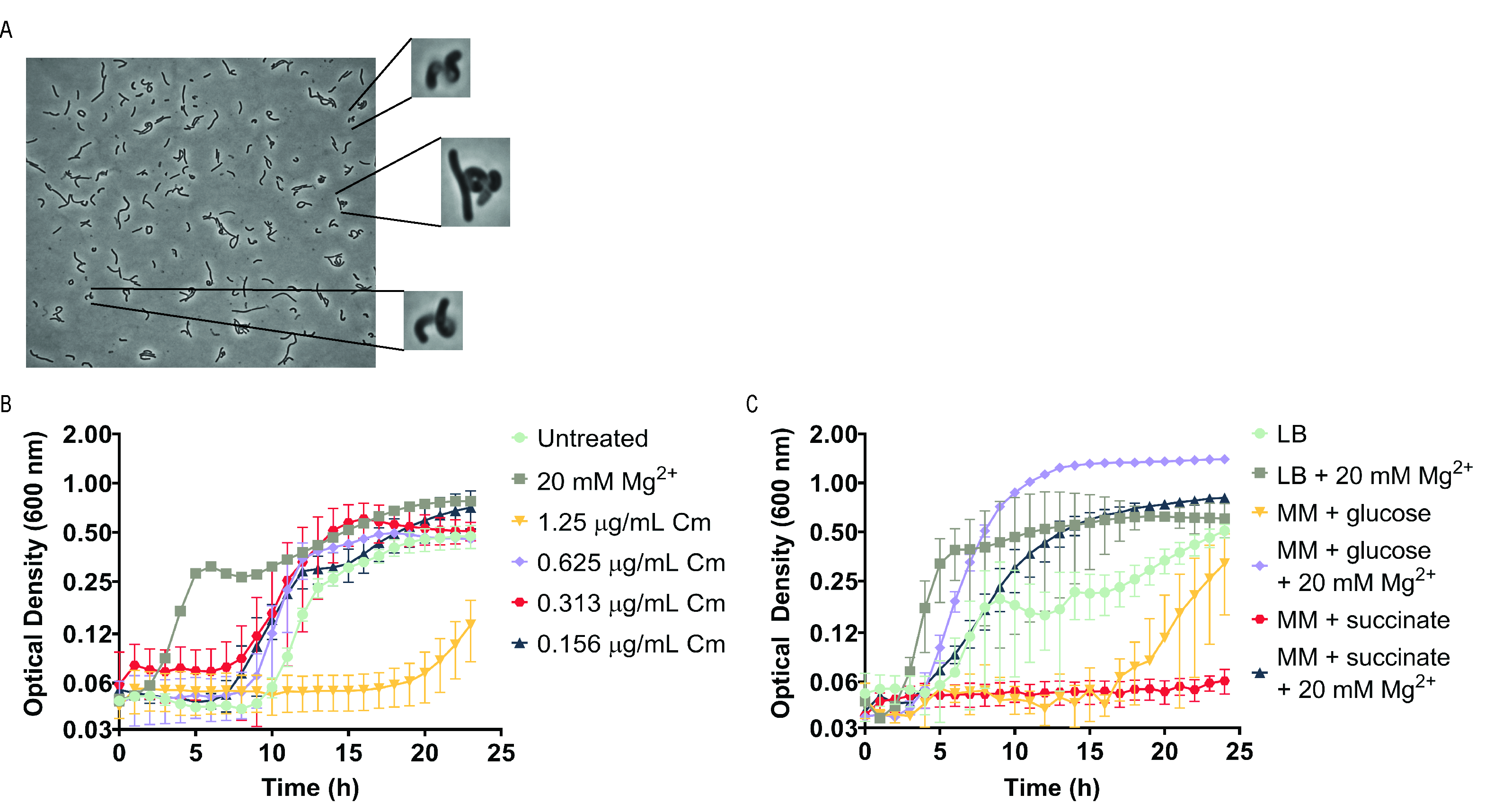

Supplement: FIG S5 [file mbio.00475-23-s0005.tif]

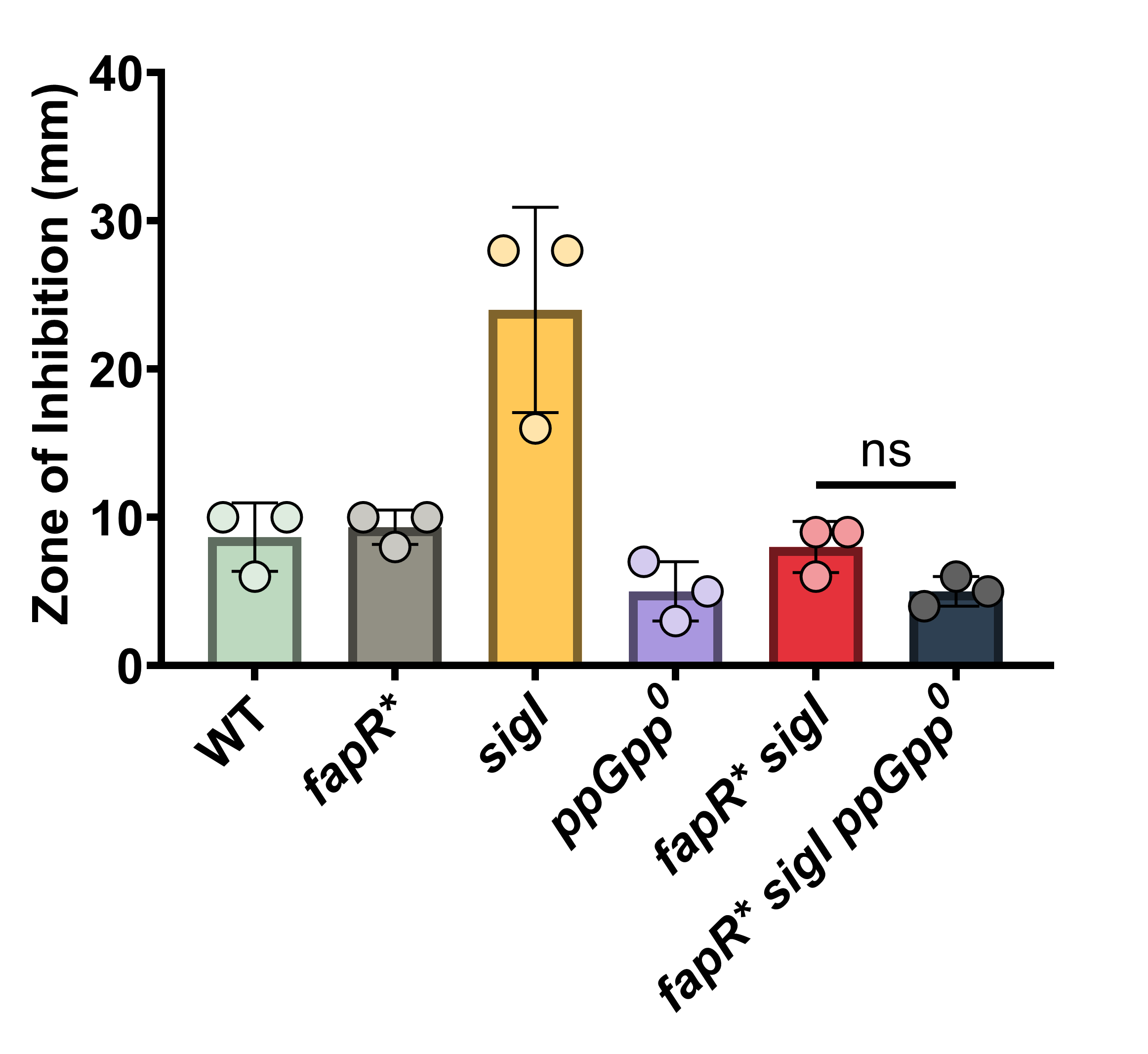

Supplement: FIG S6 [file mbio.00475-23-s0006.tif]

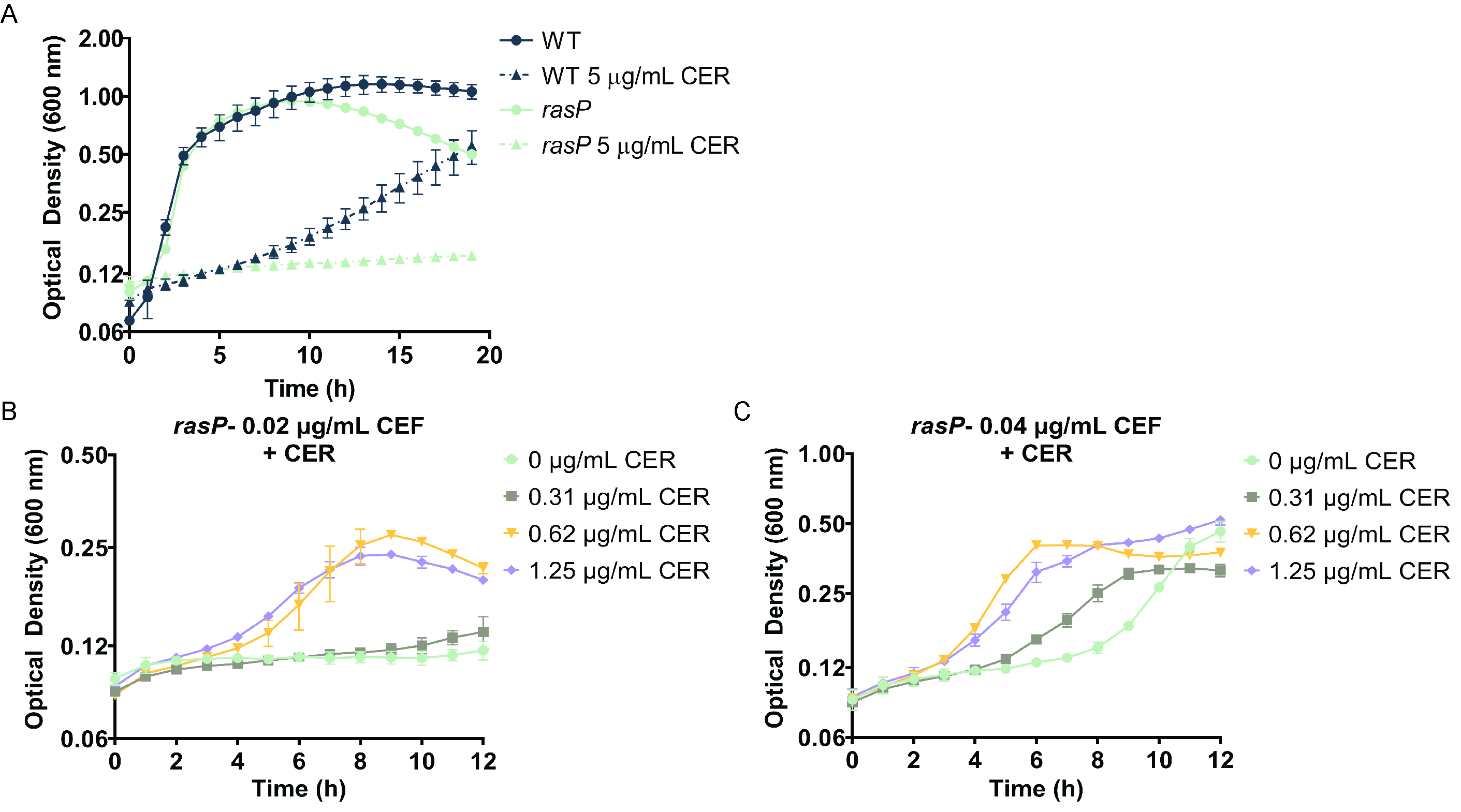

Supplement: FIG S7 [file mbio.00475-23-s0007.tif]
