# Supplementary material for: A Decrease in Fatty Acid Synthesis Rescues Cells with Limited Peptidoglycan Synthesis Capacity
Source: mBio. 2023 Apr 5;14(2):e00475-23. doi: 10.1128/mbio.00475-23 (PMC10128001; doi:10.1128/mbio.00475-23)
Supplement: TABLE S2 [file mbio.00475-23-s0009.docx]

**Table S2. The *sigI ponA* strain forms suppressors in the PG-synthesis regulating *walH*.** The suppressor mutations were identified via whole genome re-sequencing in *sigI ponA* cells that were able to grow in LB medium in the absence of added Mg^2+^. The genome position was determined via comparison to the *B. subtilis* reference genome NC_000964.3.

| Strain Background | Gene | Genome Position | Nucleotide Change | Mutation |
| --- | --- | --- | --- | --- |
| *sigI ponA* | *walH* | 4150577 | C 🡪 T | Trp429* |
| *sigI ponA* | *walH* | 4150806 | A 🡪 deletion | Val353fs |
