# Supplementary material for: A Decrease in Fatty Acid Synthesis Rescues Cells with Limited Peptidoglycan Synthesis Capacity
Source: mBio. 2023 Apr 5;14(2):e00475-23. doi: 10.1128/mbio.00475-23 (PMC10128001; doi:10.1128/mbio.00475-23)
Supplement: TABLE S3 [file mbio.00475-23-s0010.docx]

**Table S3. Strains and primers used in this study**

| **Strains** | | | |
| --- | --- | --- | --- |
| **Strain Number** | **Genotype** | **Construction** | **Reference** |
| ***B. subtilis*** |  |  |  |
| 168 | *trpC2* | Lab Strain | Lab stock |
| HB27097 | *trpC2 ΔecsA null ponA::erm* | Lab Strain | Patel et al., 2020 |
| HB27359 | *trpC2 ΔrasP null ponA::erm* | Lab Strain | Patel et al., 2020 |
| HB2044 | *trpC2 sigI::erm* | BGSC | Lab stock |
| HB27137 | *trpC2 ΔsigI null* | HB20406 --> pDR244 | This study |
| HB27108 | *trpC ponA::kan* | BGSC | Lab stock |
| HB27157 | *trpC2 ΔsigI null ponA::kan* | gDNA HB27157 -->HB27137 | This study |
| HB27065 | *trpC2 fapR** | CRISPR (see methods) | This study |
| HB25401 | *trpC2 ΔecsA null ponA::erm fapR** | SPP1 transduction *fapR** CRISPR plasmid -->HB27097 | This study |
| HB25402 | *trpC2 ΔrasP null ponA::erm fapR** | SPP1 transduction fapR* CRISPR plasmid --> HB27359 | This study |
| HB27139 | *trpC2 fapR* ΔsigI null* | HB27065 --> gDNA HB2044 --> pDR244 | This study |
| HB27158 | *trpC2 fapR* ΔsigI null ponA::kan* | HB27139 --> gDNA HB27108 | This study |
| HB27242 | *trpC2 fapR::erm* | BGSC | Lab stock |
| HB27064 | *trpC2 ΔfapR null* | HB27242 --> pDR244 | This study |
| HB27169 | *trpC2 ΔsigI null fapR::erm* | HB27137 --> gDNA HB27242 | This study |
| HB27207 | *trpC2 ΔsigI null fapR::erm ponA::kan* | HB27169 --> HB27108 | This study |
| HB27297 | *trpC2 amyE::P_spac(hy)_-fapR* | 168 --> pPL82-*fapR* | This study |
| HB27298 | *trpC2 amyE::_Pspac(hy)_-fapR** | 168-->pPL82-*fapR** | This study |
| HB27214 | *trpC2 ΔsigI null amyE::P_spac(hy)_-yqhY* | HB27137 --> pPL82-*yqhY* | This study |
| HB21116 | *trpC2 ΔsigW::erm* | Lab Strain | Lab stock |
| HB27166 | *trpC2 ΔsigW* | HB21116--> pDR244 | This study |
| HB27134 | *trpC2 ΔsigW fapR** | HB27065 --> gDNA HB21116 --> pDR244 | This study |
| HB27337 | *trpC2 ΔsigI null ΔsigW null* | HB27137 --> gDNA HB21116 --> pDR244 | This study |
| HB27338 | *trpC2 ΔsigI null ΔsigW null ponA::kan* | HB27337 --> gDNA HB27108 | This study |
| HB27173 | *trpC2 fabI::erm* | BGSC | Lab stock |
| HB27174 | *trpC2 fabL::erm* | BGSC | Lab stock |
| HB27175 | *trpC2 fabI::erm fapR** | HB27065 --> gDNA HB27173 | This study |
| HB27177 | *trpC2 fabL::erm fapR** | HB27065 --> gDNA HB27174 | This study |
| HB27230 | *trpC2 relA::erm* | BGSC | This study |
| HB27231 | *trpC2 sasA::kan sasB::tet* | gift of Heather Faega | Lab stock |
| HB27250 | *trpC2 relA::erm sasA::kan sasB::tet (ppGpp^0^)* | HB27230 --> gDNA HB27231 | This study |
| HB27233 | *trpC2 ΔsigI null fapR* relA::erm* | HB27139 --> gDNA HB27230 | This study |
| HB27240 | *trpC2 ΔsigI null fapR* relA::erm sasA::kan sasB::tet (ppGpp^0^)* | HB27233 --> gDNA HB27231 | This study |
|  |  |  |  |
| ***E. coli*** |  |  |  |
| N/A | *BL21 DE3 pET-16b-FapR* | See Methods | This study |
| N/A | *BL21 DE3 pET16b-FapR** | See Methods | This study |
|  |  |  |  |
| **Primers** |  |  |  |
|  | **Primer Name** | **Sequence** | **Purpose** |
|  | BKK-Kan-Check-R | GCAGGAGACATTCCTTCCGT | Cassette placement check |
|  | BKE MLS check R | TTTTCTCGTTCATAGTAGTTCCTCC | Cassette placement check |
|  | BKE MLS check F | CCTTAAAACATGCAGGAATTGACG | Cassette placement check |
|  | ponA F | CAAGACCTCTTTCCCCCTGC | Deletion confirmation |
|  | ecsA-check-F | TGATGTCCAGAACCCTGTCTC | Deletion confirmation |
|  | rasP-check-F | AGTAGCTGTCGCTGCCTTTT | Deletion confirmation |
|  | F-PsigI-EcoRI | ATCGAATTCCGGTTTGCGGCTGGTTTATG | Deletion confirmation |
|  | SigI-Check-Rev-JRW | CTTCATAATCGCTGTTTAACGC |  |
|  | fapR-d8-gRNA-F | tacgAACAGGAGCACGTGTTCAGC | CRISPR guide RNA cloning |
|  | fapR-d8-gRNA-R | aaacGCTGAACACGTGCTCCTGTT |  |
|  | fapR-d8-repair-up-F | AAGGCCAACGAGGCCGTCGGAGACCAATGACGGTT | CRISPR repair template cloning |
|  | fapR-d8-repair-down-R | AAGGCCTTATTGGCCTCCCGAAACAGTCGGAAGTG |  |
|  | pJOE8999-check-F | CCTTTTTGCGTGTGATGCGA | CRISPR plasmid check |
|  | pJOE8999-check-R | GTCAGCTAGGAGGTGACTGA |  |
|  | fapR-int-F | AGAAATAAGAGAGAACGCCAGGA | *fapR** sequencing confirmation |
|  | fapR-int-R | TTCGCCAACGTAGCTGTTCA |  |
|  | fapR-check-F | GACGGTTTCGAGCTGTCTGA | Deletion confirmation |
|  | fapR-check-R | ACCTCCTGCGCCATAAGAAC |  |
|  | fapR-HindIII-F | atcgaagcttAGTCTTAATTGTCCGGATGGT | *fapR/fapR** cloning into pPL82 |
|  | fapR-XbaI-R | atcgtctagaTGCATCTACAGCTATTCTCATGCA |  |
|  | pPL82-check-F-HZ | AAGAAAGATATCCTAACAGCACA | pPL82 cloning check |
|  | pPL82-check-R-HZ | ACGATCTTTCAGCCGACTCA |  |
|  | FabF-RT-Fwd-JRW | AGGAGCAAAAGGGGTGAACT | RT-PCR |
|  | FabF-RT-Rev-JRW | GTGACCATCACGTCTGCATC |  |
|  | FapR-RT-Fwd-JRW | ATTTGCACAGGCGAATTCTT | RT-PCR |
|  | FapR-RT-Rev-JRW | TTTGCTACGACACGTTCACC |  |
|  | fabHA-RT-F | GCTGGAATACTTGGTGTTGGAC | RT-PCR |
|  | fabHA-RT-R | GCTGCAACAGCCATATGTGA |  |
|  | gyrA-RT-F | GGCGGCCATGCGTTATACAG | RT-PCR |
|  | gyrA-RT-R | GCCATACCTACCGCAATGCC |  |
|  | fabI-check-F-JRW | CGCTTTTCATCTGGACAAGG | Deletion confirmation |
|  | fabI-check-R-JRW | CGGGATTGCTTGATATAGATCC |  |
|  | fabL-check-F-JRW | CCGACATACATAACAGAAGACC | Deletion confirmation |
|  | fabL-check-R-JRW | GTGCAGGTGATCGTATAGC |  |
|  | FapR-NdeI-pET-JRW | ATGCCATATGagaagaaataagagagaacgcc | *fapR/fapR** cloning into pET-16b |
|  | fapR-BamHI-pET-JRW | ATGCGGATCCTTAttatgaatgttttgaacgatacatgtc |  |
|  | pET16b-Check-F-JRW | CCTCCTTTCAGCAAAAAACC | pet-16b cloning check |
|  | pET16b-Check-R-JRW | GCGGATAACAATTCCCCTC |  |
|  | pFabHAF-L-FAM-Fwd-JRW | /56-FAM/CAAGCTCCTTTAAAGCGGG | FAM-Labeled EMSA Probe |
|  | pFabHAF-L-FAM-Rev-JRW | /56-FAM/CAGATACAAGTGCTGACCG |  |
|  | PsigW-XbaI-F | ATCGTCTAGAACTTTGACTCCGTCATGCGT | Deletion confirmation |
|  | sigW-check-R | AAGATTCGGCTGCTTGGACA |  |
|  | yqhY-HindIII-Fwd | atgcaagcttCGGAGGTGAATTGAATGAAAG | *yqhY* cloning into pPL82 |
|  | yqhY-xbaI-Rev | atgctctagaGGTTTCGTGTTAAGCCATTTAC |  |
|  | relA-Check-Fwd-JRW | CGAGCTTTCTTACCTTGACG | Deletion confirmation |
|  | relA-Check-Rev-JRW | GTATCATCGTGAGTGATGCC |  |
|  | sasA-UpCheck-JRW | CGCAAAAAGAAAGCATGGG | Deletion confirmation |
|  | sasA-DownCheck-JRW | GGGCTATCAAAAGGACTTTACC |  |
|  | sasB-UpCheck-JRW | GAATTGCTGAAGCAGCTTTTC | Deletion confirmation |
|  | sasB-DownCheck-JRW | CTTCACGATAAGAAGATCTCCC |  |
